# Salivary IFN-γ/IL-4 Ratio as a Mirror of Patient Experience: Unraveling Self-Reported Health and Disease Etiology in Sjögren’s Disease—A Pilot Study

**DOI:** 10.3390/jcm15218337

**Published:** 2026-10-26

**Authors:** Vilius Kontenis, Auksė Zinkevičienė, E. Marion Schneider, Rita Vilienė, Jūratė Gruodė, Diana Mieliauskaitė

**Affiliations:** 1 Department of Personalised Medicine, State Research Institute Center for Innovative Medicine, Santariškių St. 5, 08406 Vilnius, Lithuania; marion.schneider@uni-ulm.de (E.M.S.); jurate.gruode@gmail.com (J.G.); diana.mieliauskaite@imcentras.lt (D.M.); 2 Department of Immunology and Bioelectrochemistry, State Research Institute Center for Innovative Medicine, Santariškių St. 5, 08406 Vilnius, Lithuania; aukse.zinkeviciene@imcentras.lt (A.Z.); rita.viliene@imcentras.lt (R.V.); 3 Department of Neurology, Ulm University Hospital, Campus North, 89081 Ulm, Germany

**Keywords:** IFN-γ/IL-4 ratio, Sjögren’s disease, non-autoimmune sicca syndrome, patient’s self-reports on the disease onset, cause and activity

## Abstract

**Background/Objectives:** To determine whether self-reports on the onset, perceived causes and activity of Sjögren’s disease (SjD) can reflect the local immune status? **Materials and methods:** This prospective study enrolled a cohort of 80 patients with sicca symptoms (dry eyes and dry mouth) referred to the outpatient department of the State Research Institute Center for Innovative Medicine between September 2024 and January 2026. The study population comprised two distinct clinical groups: 37 patients diagnosed with SjD and 43 patients with non-autoimmune sicca syndrome (nonautoimmunSicca) due to gastroesophageal reflux disease (GERD). All patients indicated the perceived causes of their disease, the symptoms at the onset of the disease, and the activity of the disease using Visual analog scale (VAS). Cytokine levels in saliva were measured using enzyme-linked immunosorbent assay (ELISA). **Results:** The IFN-γ/IL-4 ratio was significantly increased in patients with SjD (median 158.7 (IQR 67.4–465.1) vs. 31.80 (IQR 20.9–45.5); *p* < 0.001). The IFN-γ/IL-4 ratio was significantly and negatively correlated with extraglandular symptoms at the onset of the disease, time from the 1st symptoms, salivary flow rates, and positively correlated with dryness score according to VAS in patients with SjD. The IFN-γ/IL-4 ratio was significantly and negatively associated with salivary flow rates and positively correlated with dryness score according to VAS in patients with nonautoimmunSicca. We also found a positive correlation between the perceived cause of the disease and the dryness score on the VAS, as well as a negative correlation between saliva flow rate and the concentration of IFN-γ in saliva among patients with SjD. The salivary IL-4 levels significantly and positively correlated with salivary flow rate, as well as negatively with pain and dryness score on the VAS in SjD group. **Conclusions:** Patient-reported information regarding disease onset, perceived causes, and symptoms severity of SjD significantly correlates with local Th1/Th2 mucosal immune status, suggesting that patient experiences may reflect underlying inflammatory trends and support patient-centered clinical evaluation.

## 1. Introduction

Sjögren’s disease (SjD) is a chronic systemic autoimmune disorder marked by glandular inflammation that impairs salivary and lacrimal function, frequently extending to multiple organ systems. Characterized by a stealthy onset, diverse clinical presentations, and unpredictable progression, it ranks among the most frequently delayed diagnoses within autoimmune pathologies [1,2]. Current research into the pathogenesis SjD increasingly adopts a multifactorial framework, suggesting that the initiation and persistence of autoimmune responses are driven by a dynamic interplay between genetic susceptibility and environmental triggers [3].

The ongoing transformation of medicine to patient-centered practice aligns with the use of patient-reported information regarding causes and conditions. These indicators provide uniquely valuable details about a patient’s condition, daily functioning, and consistent response to treatment. Furthermore, these measures have been shown to enhance communication between patients and healthcare professionals and facilitate jointly made decisions. Integrating patient-reported information into clinical management is a crucial component in advancing patient-centered, value-based healthcare provision. On the other hand, the results of studies on SjD emphasize the necessity of providing complete, patient-centered management and applying treatment approaches that address both the symptoms and the root systemic disease [4,5].

While subjective patient-reported data in SjD may align with objective clinical findings, experimental evidence reveals that salivary gland epithelial cells function as active drivers rather than passive targets of the immune response. Through the expression of immunoregulatory surface markers and the release of various cytokines and chemokines, these cells actively direct the infiltration and subsequent stimulation of leukocytes within targeted tissues [6]. The balance between salivary IFN-γ and IL-4 provides a quantifiable measure of systemic immune homeostasis, serving as a potential biomarker for chronic psychological stress, sleep disturbances, and specific oral inflammatory pathologies [7,8,9,10]. Although the baseline difference in this Th1/Th2 cytokine ratio between SjD and non-Sjögren sicca has been documented [11], previous research has predominantly focused on its correlation with objective parameters, such as salivary flow rates and focus scores. Crucially, it remains unknown whether local immune status—specifically the salivary IFN-γ/IL-4 ratio—reflects patient-reported outcome measures. Furthermore, the potential dynamic shifts of this immunological imbalance across different clinical onset types, throughout the disease timeline, and in comparison, to specific non-autoimmune sicca (nonautoimmunSicca) etiologies like gastroesophageal reflux disease (GERD) remain uncharacterized.

Therefore, the objective of this study was to determine whether self-reports on the onset, perceived causes and activity of SjD can reflect the local immune status?

## 2. Materials and Methods

### 2.1. Participants and Diagnostic Criteria

This prospective study enrolled a cohort of 80 patients manifesting sicca symptoms (ocular and oral dryness) referred to the outpatient department of the State Research Institute Center for Innovative Medicine between September 2024 and January 2026. The study population comprised two distinct clinical groups: 37 patients diagnosed with SjD and 43 patients presenting with nonautoimmunSicca due to GERD.

The SjD cohort comprised patients diagnosed in accordance with the 2016 American College of Rheumatology/European League Against Rheumatism (now ‘European Alliance of Associations for Rheumatology’) (ACR/EULAR) Sjögren’s syndrome (SS) classification criteria [12]. All patients included in the SjD group were robustly diagnosed based on positive anti-Ro/SSA serology and positive minor salivary gland histopathological examination (focus score ≥ 1). Conversely, the comparison group consisted of individuals with newly diagnosed, nonautoimmunSicca due to GERD, who presented with symptoms of dry eyes and/or dry mouth. In the comparison group, a board-certified gastroenterologist-endoscopist confirmed the presence of GERD through a comprehensive evaluation of the patients’ clinical symptoms and esophagogastroduodenoscopy findings based on standard clinical guidelines. Upper gastrointestinal endoscopy was performed in all 43 patients. *H. pylori* infection status was routinely assessed during endoscopy via rapid urease test and stool antigen test. Patients with active, untreated *H. pylori* infection or those undergoing current eradication therapy were excluded from the study to prevent potential confounding effects on local cytokine production.

Screening for sicca features, testing for anti-SSA(Ro) seropositivity, and performing histopathological assessments for focal lymphocytic sialadenitis (focus score ≥ 1 focus/mm^2^) were mandatory for all control subjects to definitively rule out any systemic autoimmune involvement. Consequently, these participants failed to meet the ACR/EULAR criteria for SS, and no alternative etiologies for sicca symptoms other than GERD were identified.

All patients indicated the perceived cause, the presenting symptomatology at disease onset, and the severity of the symptoms using Visual analog scale (VAS).

Notably, all study participants across both cohorts were treatment-naive for their respective conditions (SjD or GERD) at the time of enrollment.

### 2.2. Saliva Collection

To ensure the validity of the cytokine measurements in saliva, the following strict exclusion criteria were applied and verified for all participants in both groups:

1. Local oral and lifestyle confounders: Patients with active periodontal disease, severe gingivitis, poor oral health status, or clinical signs of oral infections (such as oral candidiasis/thrush or active viral lesions) were strictly excluded. Compliance with this criterion was verified via a standardized clinical oral cavity examination performed by a specialist prior to saliva collection. Furthermore, all included patients were non-smokers, eliminating the confounding effects of tobacco use on mucosal immunity.
2. Systemic immunomodulatory confounders: Patients with any history of other major concurrent autoimmune or chronic inflammatory diseases, or acute infections within 4 weeks of enrollment, were excluded. Most importantly, none of the included patients were taking systemic immunosuppressive medications (including systemic corticosteroids, biologics, or disease-modifying antirheumatic drugs) or regular non-steroidal anti-inflammatory drugs at the time of sampling or within the preceding 4 weeks.

To perform unstimulated sialometry, patients must refrain from eating, drinking, or oral hygiene activities for at least two hours prior to the test. Before commencing the collection, patients are instructed to swallow any residual saliva to empty the oral cavity. During the procedure, the subject is maintained in an erect seated position with the cranium inclined slightly anteriorly, facilitating the spontaneous accumulation of saliva. Saliva is collected over a continuous 15-min period. The patient accumulates saliva in the floor of the mouth and expectorates it into a specialized collection tube via a funnel, without swallowing or stimulating the salivary glands. Following the 15 min collection period, the total volume of the sample is measured and recorded as the salivary flow rate in milliliters per 15 min (mL/15 min).

Samples with visible blood contamination were excluded, and none of the analyzed samples showed signs of contamination. The collected saliva was stored at −80 °C and thawed at 4 °C immediately before analysis.

### 2.3. Cytokine Analysis

Quantification of salivary cytokine concentrations was performed utilizing commercially available enzyme-linked immunosorbent assay (ELISA) kits, strictly adhering to the protocols provided by the manufacturer. The specific kits used were the Human IFN-γ ELISA Kit (Cat. #KHC4022) and the IL-4 Human High Sensitivity ELISA Kit (Cat. #BMS225HS), both from Invitrogen (Thermo Fisher Scientific, Waltham, MA, USA). All samples were measured in duplicate within a single assay run.

### 2.4. Statistical Analysis

Continuous metrics with a normal distribution are reported as mean ± standard deviation (SD), while skewed distributions are characterized by the median and its corresponding first and third quartiles (Q1–Q3), with normality status determined via the Shapiro–Wilk approach. To evaluate differences between groups, the independent samples *t*-test was applied to normally distributed variables, whereas non-parametric data were analyzed via the Mann–Whitney U test. Categorical metrics were assessed using Fisher’s exact test. Furthermore, the relationships between clinical features and cytokine concentrations were quantified using Spearman’s rank correlation coefficient (r). The magnitude of the correlation coefficients was categorized into three tiers: weak (|r| < 0.30), moderate (0.30 ≤ |r| < 0.60), and strong (|r| ≥ 0.60). Statistical significance was established at a two-tailed *p*-value of < 0.05. All computational and statistical evaluations were executed utilizing SAS software (version 9.2). SAS Institute Inc., (Cary, NC, USA).

## 3. Results

### 3.1. Characteristics of the Study Groups

The investigation cohort comprised 37 individuals diagnosed with SjD, exhibiting a mean age of 52.8 years (SD = 9.78; SE = 1.61), alongside a reference cohort of 43 patients presenting with nonautoimmunSicca due to GERD (mean age: 51.7 years; SD = 9.65; SE = 1.46). In the SjD cohort, sicca onset was observed in 8 patients (21.62%), whereas 29 patients (78.38%) presented with extraglandular onset symptoms. Conversely, the most of patients with nonautimmunSicca due to GERD experienced sicca onset (*n* = 34; 79.07%). Psychological stress was identified as the primary triggering factor in the SjD group (*n* = 23; 62.16%). In contrast, patients with nonautoimmunSicca due to GERD predominantly attributed their symptoms to dietary habits or reported an unknown etiology in equal proportions (20 patients; 46.5%) while three patients (7.0%) identified stress as a trigger.

Furthermore, the of SjD reported greater fatigue, pain, and overall dryness on the visual analogue scale (Figure 1).

### 3.2. Cytokine Levels in Saliva of Study Groups

A significant variance in IL-4 concentrations was observed when comparing the SjD cohort to the non-autoimmune sicca group (median 0.20 (IQR 0.20–0.40) vs. 0.4 (IQR 0.20–0.60) pg/mL; *p* = 0.024). IFN-γ concentrations were significantly higher in the SjD (median 53.2 (IQR 19.4–90.3) pg/mL;) compared with nonautoimmunSicca group (12.7 (medianIQR 7.4–21.1) pg/mL; *p* < 0.001). Consequently, the IFN-γ/IL-4 ratio was also significantly increased in patients with SjD (median 158.7 (IQR 67.4–465.1) vs. 31.80 (IQR 20.9–45.5); *p* < 0.001) (Table 1, Figure 2A–C). Also, our findings demonstrate a marked reduction in salivary secretory capacity among individuals diagnosed with SjD. (Table 1, Figure 2C).

### 3.3. Associations Between Cytokine Levels in Saliva and Clinical Characteristics

An inverse significant correlation was observed between the salivary IFN-γ/IL-4 ratio and disease onset extraglandular symptoms, time from the 1st symptoms, salivary flow rates, and positively associated with dryness score according to VAS in patients with SjD. We also found a positive correlation between the reported perceived cause of the disease and the dryness score on the VAS, as well as a negative correlation between saliva flow rate and the concentration of IFN-γ in saliva within the SjD cohort D. The salivary IL-4 levels significantly and positively correlated with salivary flow rate, as well as negatively with pain and dryness score on the VAS in SjD group (Figure 3).

A significant negative association was established between IFN-γ/IL-4 ratio and. salivary flow rates, and IFN-γ/IL-4 ratio was positively associated with dryness score according VAS in patients with nonautoimmunSicca. The IFN-γ levels in saliva were independently and negatively associated with salivary flow rates in nonautoimmunSicca patients (Figure 4).

When analyzing all study participants, we found a negative association between IL-4 and pain and dryness scores on the VAS, as well as a negative association between IFN-γ and salivary flow and a positive association with pain and dryness scores on the VAS. Meanwhile, the salivary IFN-γ/IL-4 ratio exhibited a negative correlation with salivary flow and positively correlated with VAS scores for fatigue, pain, and dryness (Figure 5).

## 4. Discussion

Owing to its stealthy onset, diverse clinical presentations, and unpredictable progression, SjD ranks among the most frequently delayed diagnoses within autoimmune pathologies [1]. A study that assessed factors related to late diagnosis of SjD demonstrated that such late diagnosis is one of the patients’ unmet needs. Efforts must be made to ensure that the disease is diagnosed at the very earliest stage [13]. Integrating patient-reported outcomes into clinical workflows is essential for advancing patient-centered, value-based healthcare. Concurrently, empirical evidence regarding SjD underscores the imperative for comprehensive, holistic patient management, necessitating therapeutic strategies that simultaneously target phenotypic symptoms and underlying systemic pathology [4,5].

Patients with SjD exhibit a unique phenotype of salivary gland epithelial cells (SGECs), which can initiate and sustain non-specific and antigen-specific immune pathways within the local glandular niche [14]. Therefore, to determine whether the causes and symptoms of the disease as described by the patients themselves reflect the actual immune status, we chose to investigate the IFN- γ/IL-4 ratio in saliva.

Our findings demonstrate that individuals with SjD present with significantly elevated salivary concentrations of the pro-inflammatory cytokine IFN-γ, alongside an increased IFN-γ/IL-4 ratio, relative to the nonautoimmunSicca due to GERD cohort. Consistent with contemporary literature, the upregulation of specific cytokines in the saliva and tear fluid of individuals with SjD underscores a robust interplay between innate and adaptive immune pathways [15].

The study, which echoes the results of our research, found that patients with SjD have elevated concentrations of Th1, Th2, and Th17 cytokines in their saliva compared to individuals in the control group who do not experience symptoms of dryness (non-sicca controls). However, there was significant overlap in cytokine concentration levels in the saliva of SjD group and those with non-Sjögren’s syndrome-related dryness (non-SS sicca). The key features that best distinguished SjD from non-SS sicca were higher TNF-α concentrations and a higher Th1/Th2 ratio. Furthermore, the Th1/Th2 ratio was found to correlate with clinical parameters of SjD [11]. Our finding regarding the baseline difference in the salivary IFN-γ/IL-4 ratio between SjD and nonautoimmunSicca aligns with well-established literature, including the landmark study [11]. However, while this study primarily focused on linking this Th1/Th2 imbalance to objective parameters like salivary flow rates and histopathological focus scores, our study expands upon these findings by bridging the gap between local immune status and patient-reported clinical phenotypes.

Specifically, we demonstrated that the local IFN-γ/IL-4 ratio is significantly associated with patient-reported initial clinical presentation, such as the type of disease onset symptoms (glandular vs. extraglandular) and the time elapsed since the first symptoms. The negative association identified between the IFN-γ/IL-4 ratio and extraglandular onset symptoms suggests that the local cytokine profile may evolve dynamically over the disease timeline, shifting as clinical manifestations change. This adds a new layer of complexity to the Th1/Th2 paradigm in SjD, suggesting that mucosal inflammation is not static but rather reflects the chronological and clinical progression perceived by the patient.

Furthermore, unlike previous cohorts that utilized healthy individuals or generic sicca controls, our study employed a highly specific differential diagnosis control group—patients with nonautoimmunSicca due to GERD induced dryness. Distinguishing SjD from GERD-related sicca is a frequent clinical challenge. By characterizing the distinct salivary cytokine profile in this specific cohort, our data offer enhanced clinical utility for biomarker-driven differential diagnosis. Finally, the negative correlation between isolated salivary IL-4 levels and patient-reported pain and dryness (VAS) underlines the potential clinical relevance of monitoring anti-inflammatory pathways, directly linking molecular biomarkers to patient comfort and symptom severity.

Our study results reveal an important insight into the relationship between the IFN-γ/IL-4 ratio (reflecting the local Th1/Th2 immune balance), salivary flow, and dryness scores. However, the underlying mechanisms driving these associations likely differ between the studied cohorts. While the correlation in SjD points to intrinsic autoimmune salivary gland dysfunction, the findings in the nonautoimmunSicca due to GERD require cautious interpretation.

In the GERD cohort, sicca symptoms and altered salivary parameters are primarily driven by chronic mucosal irritation from gastric refluxate, such as acid and pepsin, rather than primary glandular pathology. Therefore, the observed association between the IFN-γ/IL-4 ratio and dryness scores in GERD patients does not imply intrinsic salivary gland dysfunction. Instead, it likely reflects a local mucosal irritation and a non-autoimmune inflammatory response to chronic reflux. Future studies are needed to further elucidate how localized neurogenic or inflammatory pathways in GERD interact with systemic immune markers like the IFN-γ/IL-4 ratio.

Our findings demonstrate that an elevated IFN-γ/IL-4 ratio is strongly associated with salivary gland hypofunction and heightened dryness perception (VAS) in both SjD and nonautoimmunSicca. These observations align with recent breakthrough data demonstrating that CD4+ T-cell derived IFN-γ can impair salivary secretion by triggering ferroptosis in epithelial cells and down-regulating aquaporin-5 (AQP5) expression. Although our study design captures statistical associations rather than direct causality, this Th1-dominant response—reflected by a higher IFN-γ/IL-4 ratio—potentially contributes to the observed lower salivary flow rates through similar interconnected pathophysiological pathways [16].

Recent single-cell transcriptomic insights substantiate that IFN-γ-associated genetic profiles and macrophage diversity are pivotal drivers of microenvironmental impairment within the salivary glands. This reinforces our findings regarding the influence of the impendent IFN-γ/IL-4 ratio on disease progression [17].

The authors of the recently published study emphasize that inflammation of the salivary glands and symptomatic features such as pain and sicca manifestations are regulated by specific cytokine pathways, including the IFN-γ–IDO (Indoleamine 2,3-dioxygenase) –kynurenine axis. This neuroimmune mechanism may well explain why, in our study, a high IFN-γ/IL-4 ratio directly correlates with a stronger subjective sensation of dryness as measured on the VAS scale [18].

Furthermore, studies show that the activated IFN-γ signaling pathway is reliably associated with the development of extraglandular manifestations (EGM) in patients with SjD. This directly supports our finding that the IFN-γ/IL-4 ratio was negatively associated with the extraglandular symptoms at the onset of disease and the time from the onset of the first symptoms [19].

The scientific literature indicates that the extraglandular (non-glandular/systemic) manifestation of SjD is the first symptom in approximately 5–15% of patients. However, in studies of specialized clinical cohorts, this figure can reach as high as 53% [20,21,22]. According to the results of our study, 78.38%% of SjD cases began with extraglandular symptoms manifestations. There is no doubt that extraglandular manifestations in the early clinical onset of SjD delay confirmation of the diagnosis, and this once again demonstrates how important it is to listen to the patient’s account of their experience with the disease.

A recent study shows that the heterogeneous clinical and serological characteristics observed in SjD patients are associated with the cytokine microenvironment [23]. A study conducted some time ago showed that the immune response changes at the onset of the disease and as it progresses (as inflammatory foci in the salivary glands expand). In the early stages, a single type (Th1) of cytokine profile dominates, but as the lesions worsen and the disease progresses, this balance may shift, involving other immune pathways as well [24]. Recent studies emphasize that it is precisely a qualitative imbalance in immune pathways (for example, changes in the activity of IFN-γ or type II interferon signaling) is strongly associated with the subjective burden experienced by patients—pain, fatigue, and a sensation of dryness—as measured by patient questionnaires [such as the EULAR Sjögren’s Syndrome Patient Reported Index (ESSPRI) or VAS] [25].

Our results indicate a clear role for Th1 and Th2 cytokine imbalance in the development of symptoms. The negative association of IL-4 with pain and dryness confirms the protective and anti-inflammatory role of this cytokine in peripheral tissues. In contrast, IFN-γ production directly contributes to the suppression of salivary gland secretory function and neuroinflammation, which manifests as reduced salivary flow and a more intense sensation of pain and dryness. Most importantly, the IFN/IL-4 ratio has been shown to be a sensitive marker of systemic inflammation, strongly correlated not only with local symptoms but also with systemic fatigue, likely due to the central effects of IFN-γ on the nervous system [26].

The principal merit of this investigation resides in its innovative nature, representing the first scientific effort to investigate whether patient-reported outcomes—specifically regarding the onset, perceived cause, and activity of SjD—can reliably reflect local immune status using a non-invasive biological matrix. By demonstrating the utility of saliva collection, this work establishes a patient-friendly approach that minimizes clinical burden while contributing to the framework of personalized management in SjD. Furthermore, a key clinical asset of this design is its pragmatic approach to differential diagnosis, focusing directly on distinguishing between autoimmune SjD and nonautoimmunSicca due to GERD in clinically symptomatic populations.

Conversely, a few limitations of the present study should be noted. First, the absence of a healthy control cohort restricts our ability to establish a baseline normative range, which would have further validated the diagnostic utility of the salivary IFN-γ/IL-4 ratio. Secondly, as this pilot study was inherently exploratory and featured a restricted cohort size, the study design was naturally geared toward screening for meaningful baseline correlations and generating novel hypotheses, rather than constructing robust multivariable predictive models. Consequently, without multi-variable regression analysis, the independent predictive value of these patient-reported associations remains to be fully confirmed. Furthermore, a limitation of this study is the omission of the EULAR Sjögren’s Syndrome Disease Activity Index (ESSDAI) score and additional serum biomarkers, which could have provided a more comprehensive evaluation of systemic disease activity and extraglandular manifestations.

To build upon these preliminary findings, several avenues for future research are warranted. To transition from hypothesis generation to robust clinical validation, larger, multi-center longitudinal studies are required. Increasing the cohort size will provide the statistical power necessary to perform multivariable regression analyses, thereby adjusting for potential confounding clinical variables and confirming the independent predictive strength of patient self-reports. Finally, longitudinal monitoring of salivary biomarkers alongside fluctuating patient-reported disease activity scores could clarify the dynamic relationship between mucosal immunity and clinical symptoms over time, paving the way for validated, non-invasive tools in routine SjD monitoring.

In summary, patient-reported information regarding disease onset, perceived causes, and symptoms severity of SjD significantly correlates with local Th1/Th2 mucosal immune status, suggesting that patient experiences may reflect underlying inflammatory trends and support patient-centered clinical evaluation.

## Figures and Tables

**Figure 1:**
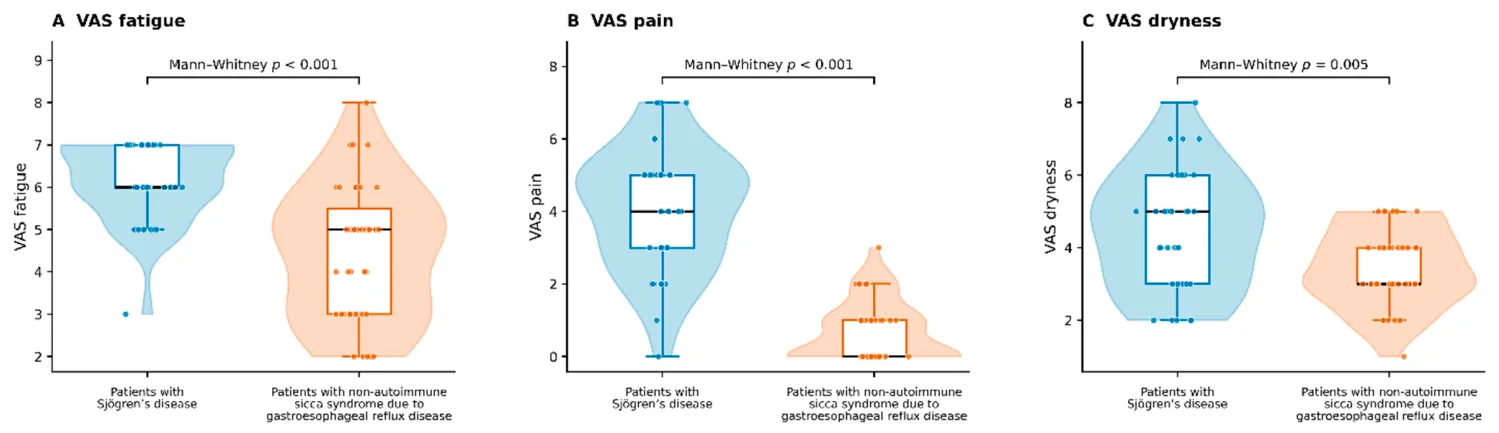
Visual analog scale (VAS) scores for (**A**) chronic fatigue, (**B**) pain, and (**C**) dryness severity across study groups.

**Figure 2:**
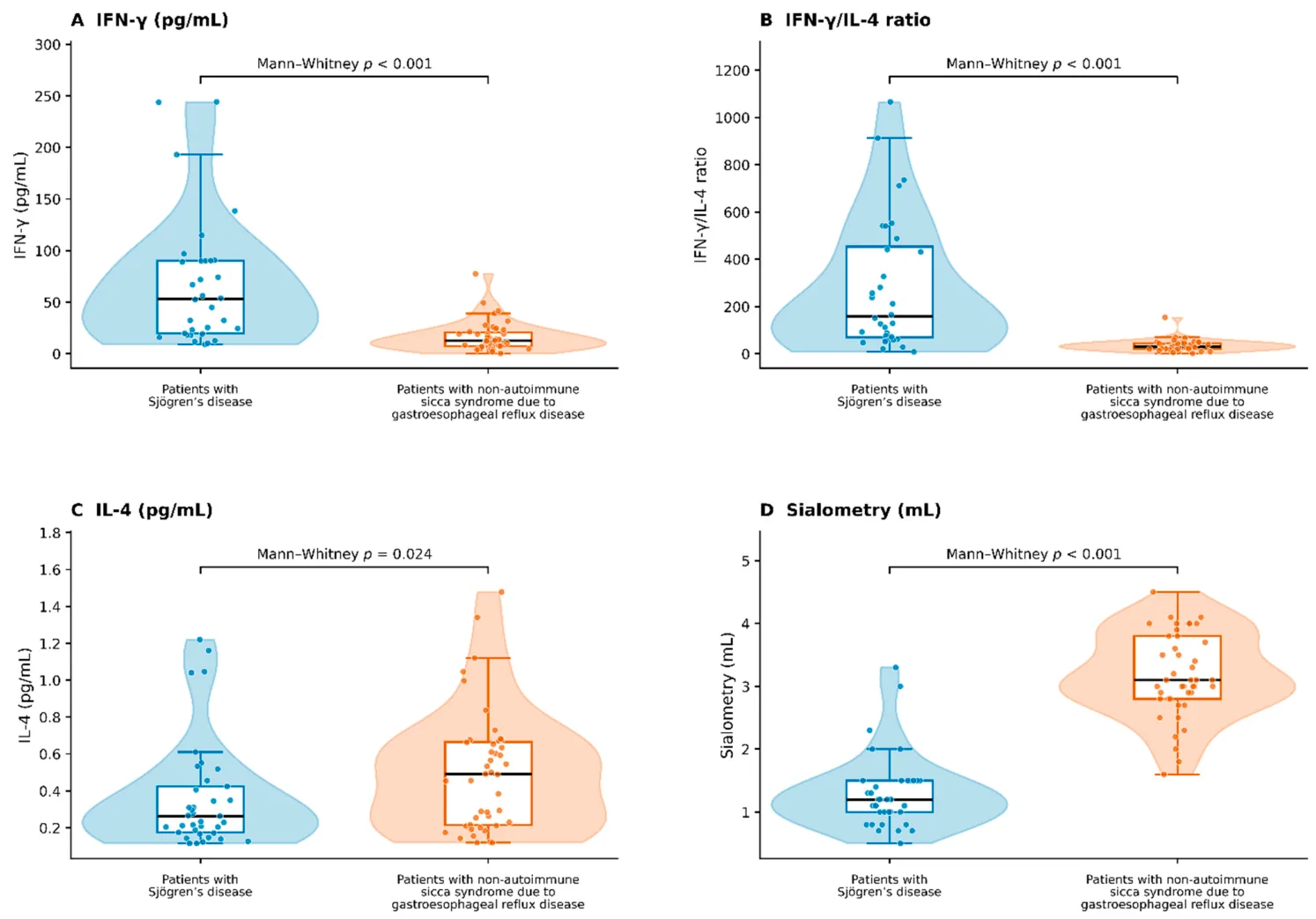
Salivary cytokine levels (**A**–**C**) and sialometry results (**D**) across the study groups.

**Figure 3:**
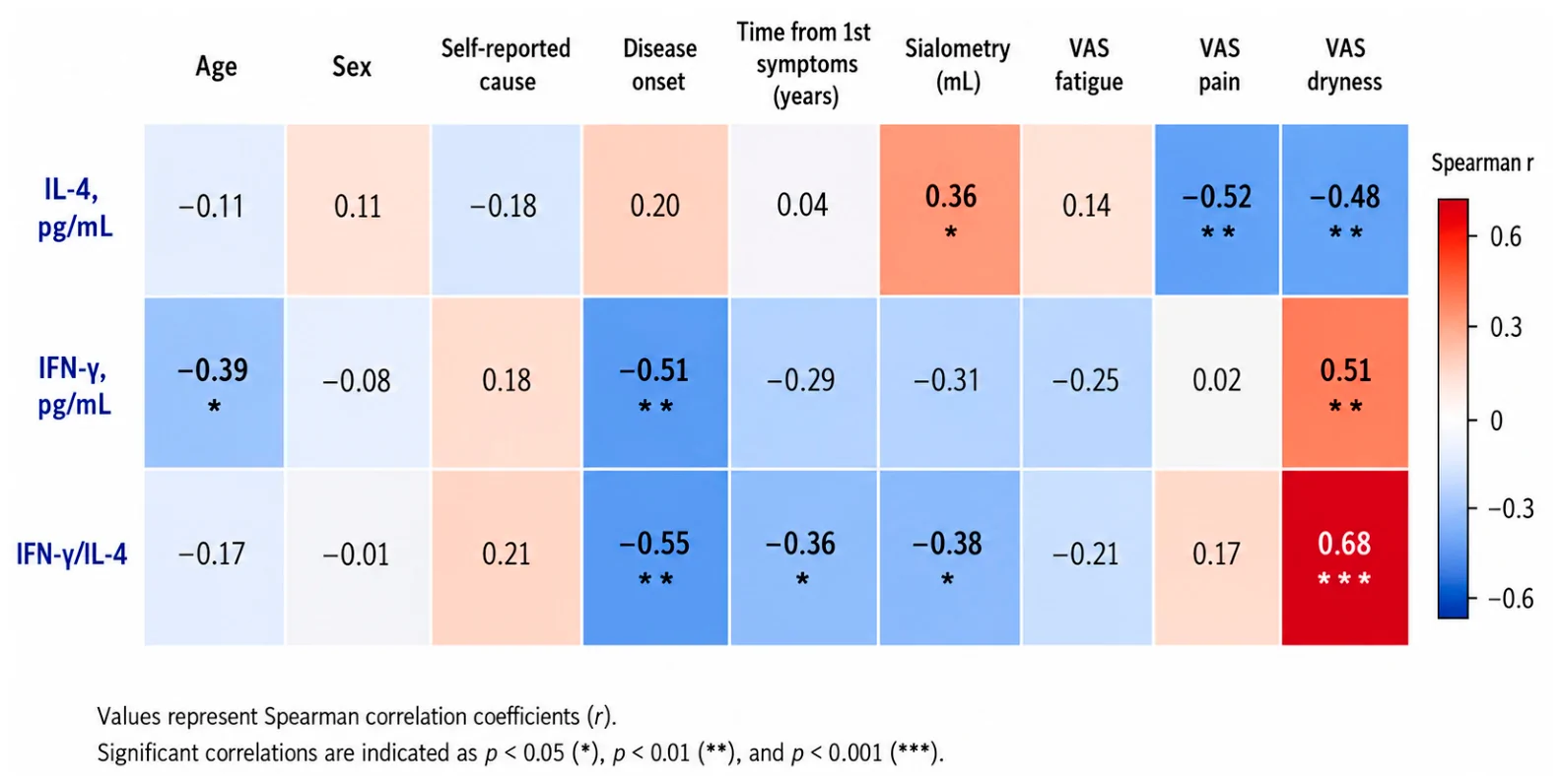
Spearman correlation matrix between salivary cytokine levels and clinical characteristics of patients with Sjögren’s disease.

**Figure 4:**
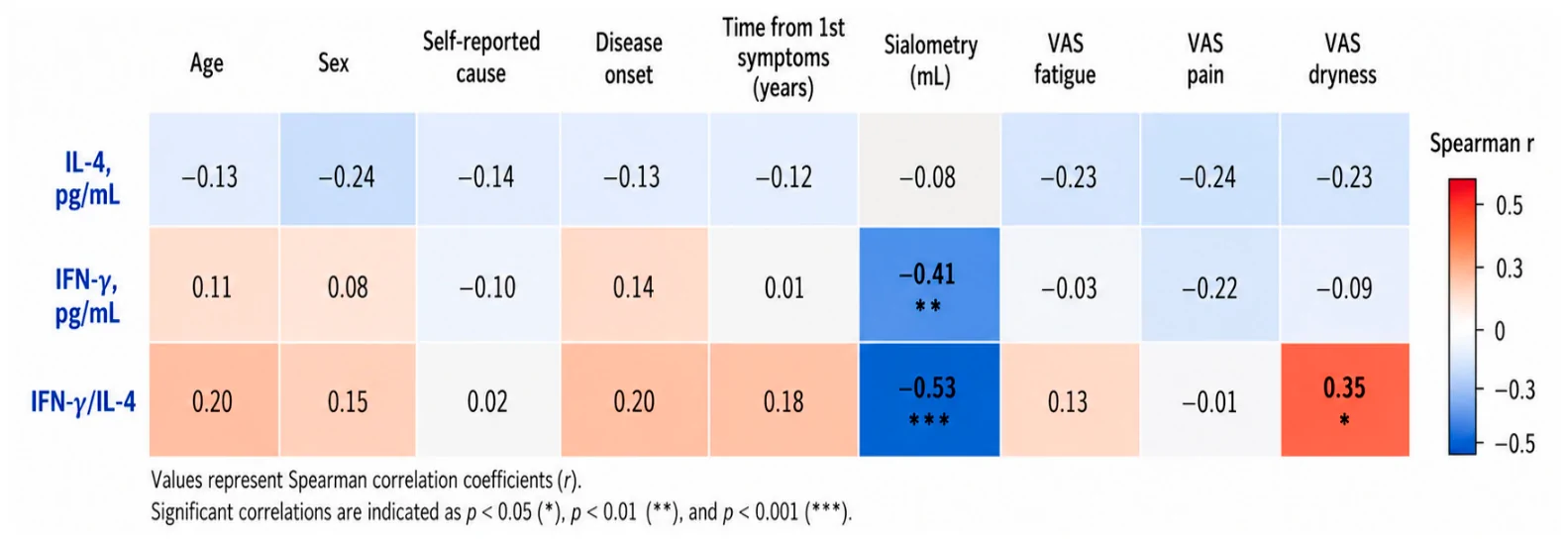
Spearman correlation matrix between salivary cytokine levels and clinical characteristics in patients with non-autoimmune sicca syndrome due to gastroesophageal reflux disease.

**Figure 5:**
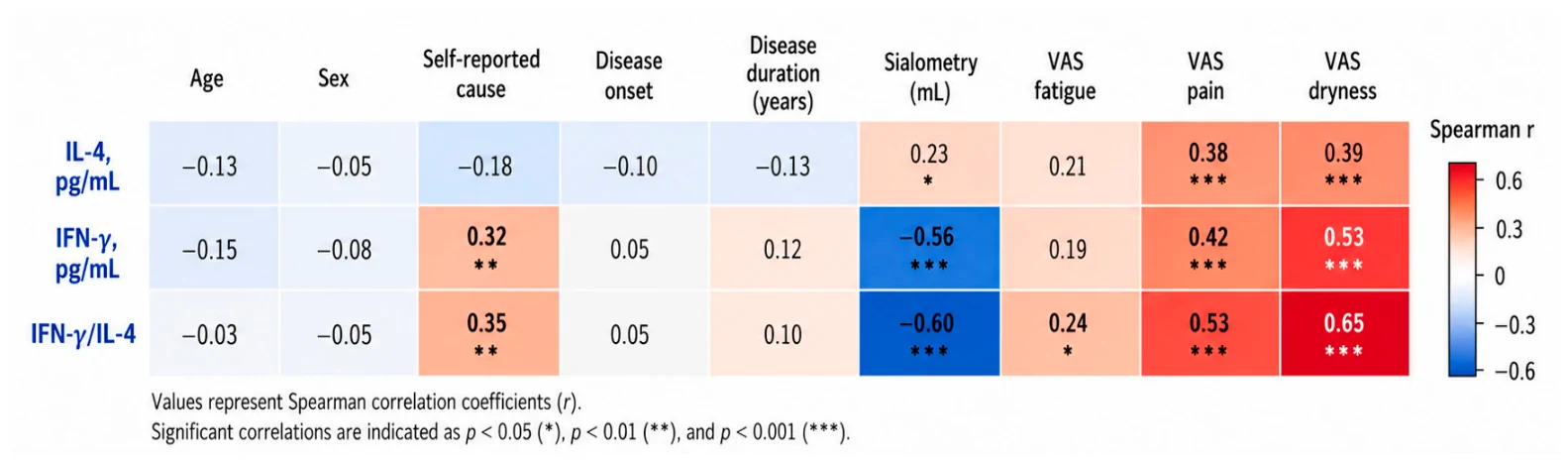
Spearman correlation Matrix between salivary cytokine levels and clinical characteristics in all study patients.

**Table 1:** Quantitative characteristics of the target groups.

Parameter	Total (*N* = 80)	Sjögren’s Disease (*N* = 37)	Non-Autoimmune Sicca Syndrome (*N* = 43)	*p*-Value
**Age** (years), Mean ± SD	52.2 ± 9.7	52.8 ± 9.8	51.7 ± 9.7	0.605 ^1^
**IL-4** (pg/mL) Mediana [Q1; Q3] Min–Max	0.40 [0.20; 0.60] 0.0–2.0	0.20 [0.20; 0.40] 0.0–2.0	0.40 [0.20; 0.60] 0.0–2.0	0.024 ^2^
**IFN-γ** (pg/mL) Mediana [Q1; Q3] Min–Max	19.1 [10.1; 49.4] 0–244	53.2 [19.4; 90.3] 9–244	12.7 [7.4; 21.1] 0–77	<0.001 ^2^
**IFN-γ/IL-4** Mediana [Q1; Q3] Min–Max	48.0 [26.3; 128.9] 3–1066	158.7 [6.4; 465.1] 8–1066	31.8 [20.9; 45.5] 3–154	<0.001 ^2^
**Sialometry** (mL) Mediana [Q1; Q3] Min–Max	2.5 [1.2; 3.1] 1–5	1.2 [1.0; 1.5] 1–3	3.1 [2.8; 3.8] 2–5	<0.001 ^2^
**Time from 1st symptoms** (years) Mediana [Q1; Q3] Min–Max	2.0 [1.0; 4.0] 1–9	4.0 [3.0; 5.0] 1–9	2.0 [1.0; 2.0] 1–4	<0.001 ^2^
**VAS, fatigue** Mediana [Q1; Q3] Min–Max	5.5 [4.0; 6.5] 2–8	6.0 [6.0; 7.0] 3–7	5.0 [3.0; 6.0] 2–8	<0.001 ^2^
**VAS, pain** Mediana [Q1; Q3] Min–Max	2.0 [0.0; 4.0] 0–7	4.0 [3.0; 5.0] 0–7	0.0 [0.0; 1.0] 0–3	<0.001 ^2^
**VAS, dryness** Mediana [Q1; Q3] Min–Max	4.0 [3.0; 5.0] 1–8	5.0 [3.0; 6.0] 2–8	3.0 [3.0; 4.0] 1–5	0.005 ^2^

SD: standard deviation; Q1: first quartile; Q3: third quartile; VAS: visual analog scale. ^1^ Calculated using Student’s *t*-test (data normally distributed). ^2^ Calculated using Mann-Whitney U test (data non-normally distributed).

## Data Availability

The data presented in this study are available on request from the corresponding author.

## Abbreviations

The following abbreviations are used in this manuscript:

SjD	Sjögren’s disease.
pSS	primary Sjögren’s syndrome.
NonautoimmunSicca	Non-autoimmune sicca syndrome due to gastroesophageal reflux disease.
IFN-γ	Interferon gamma.
IL-4	Interleukin-4.
Th1	Type 1 helper T cell.
Th2	Type 2 helper T cell.
VAS	Visual analog scale.
GERDWe	Gastroesophageal reflux disease.
ACR-EULAR	American College of Rheumatology/European League Against Rheumatism (now ‘European Alliance of Associations for Rheumatology).
